# The Role of Potassium Channels in *Arabidopsis thaliana* Long Distance Electrical Signalling: AKT2 Modulates Tissue Excitability While GORK Shapes Action Potentials

**DOI:** 10.3390/ijms19040926

**Published:** 2018-03-21

**Authors:** Tracey Ann Cuin, Ingo Dreyer, Erwan Michard

**Affiliations:** 1 Tasmanian Institute of Agriculture, University of Tasmania, Hobart, TAS 7001, Australia; 2 SupAgro Montpellier, 2, Place Viala, 34060 Montpellier, France; 3 Centro de Bioinformática y Simulación Molecular (CBSM), Universidad de Talca, 2 Norte 685, 3460000 Talca, Chile; 4 Cell Biology and Molecular Genetics, Biosciences Research Building, University of Maryland, College Park, MD 20742, USA

**Keywords:** action potentials, membrane transport and excitability, potassium channels, mathematical modelling

## Abstract

Fast responses to an external threat depend on the rapid transmission of signals through a plant. Action potentials (APs) are proposed as such signals. Plant APs share similarities with their animal counterparts; they are proposed to depend on the activity of voltage-gated ion channels. Nonetheless, despite their demonstrated role in (a)biotic stress responses, the identities of the associated voltage-gated channels and transporters remain undefined in higher plants. By demonstrating the role of two potassium-selective channels in *Arabidopsis thaliana* in AP generation and shaping, we show that the plant AP does depend on similar *Kv*-like transport systems to those of the animal signal. We demonstrate that the outward-rectifying potassium-selective channel GORK limits the AP amplitude and duration, while the weakly-rectifying channel AKT2 affects membrane excitability. By computational modelling of plant APs, we reveal that the GORK activity not only determines the length of an AP but also the steepness of its rise and the maximal amplitude. Thus, outward-rectifying potassium channels contribute to both the repolarisation phase and the initial depolarisation phase of the signal. Additionally, from modelling considerations we provide indications that plant APs might be accompanied by potassium waves, which prime the excitability of the green cable.

## 1. Introduction

Fast and ubiquitous signals are vital in the response of an organism to any external threat, challenge or stimulus. In animals, electrical signals such as action potentials (APs) transmit information from one part of the body to another via nerve cell axons connected by synapses. Plants also show electrical responses, occurring after stimuli such as abrupt changes in temperature and light intensity, the action of depolarising agents (potassium, glutamate etc.), wounding and the application of electrical currents [1,2]. The stimulation of a plant tissue is thought to modify the activities of plasma membrane ion channels/transporters, which in turn, affects the membrane voltage. There are several types of electrical responses in plants: local electrical responses of cells in the immediate environment of a stimulus, local periodic electrical oscillations and propagating electric signals. The latter further differentiate in action potentials (APs), variation potentials (VPs) and system potentials [3,4].

In short, VPs are long-term—in the minute range. The depolarisations are characterised by an irregular shape, which can be accompanied in many cases, by additional depolarisation voltage spikes. They are induced by local damage, for instance, local burning or wounding. As they can pass through dead tissues, they are thought to be secondary effects of hydraulic and/or chemical signals. Variation potential intensity and duration are proportional to the intensity of the damaging stimulus, and signals decrease with increasing distance from the damaged zone.

In contrast, action potentials (APs) are induced by non-damaging stress such as cooling, touch or electric currents. They only propagate through living tissues and share similar characteristics to those of animals APs, namely (i) an apparent all-or-nothing character, (ii) a constant amplitude and duration under control conditions and (iii) self-propagation [5,6,7]. Compared to animal APs however, propagation velocities and other dynamical parameters of plant APs are far slower, from millisecond duration and 1 m/s velocity in animals but >10 s duration and 1 mm/s propagation speed in Arabidopsis. Nonetheless, the self-propagating nature of plant APs suggests that, as in animal cells, transmission depends on the fluxes of ions through voltage-gated channels. However, no physiological or genetic evidence has been offered so far to validate this hypothesis.

Experimental data from Characean algae [8,9,10] and diverse modelling approaches in plants have suggested some mechanistic details for plant APs [3,7,11,12,13,14,15,16,17,18]. Experimental data suggest that the AP signal is initiated by a passive influx of calcium, an inhibition of the plasma membrane H^+^-ATPase activity and an anion efflux, which results in depolarisation of the cell. A subsequent potassium efflux has been proposed to repolarise the cell. So far though, the only molecular players that have been identified in plant AP initiation are from the glutamate receptors channel family: GLR3.2, GLR3.3 and GLR3.6 [19]. Although these GLRs have not been characterised, other members from this family have been shown to form non-selective Ca^2+^-permeable channels at the plasma membrane [20,21,22,23].

However, the molecular identity of the channels involved in the depolarisation and repolarisation phases of the membrane during the passage of an AP in higher plants are still open to conjecture. Non-selective Ca^2+^-permeable channels such as GLRs [19,22], and anion release through voltage-dependent R-type anion channels, such as QUAC1 (QUickly activating Anion Channel 1) [24,25], or S-type Ca^2+^-dependent and voltage-dependent SLAC1 (SLowly activating Anion Channel 1) [26] are good candidates for the initial depolarisation. Subsequently, P-type proton ATPase from the AHA (Arabidopsis H^+^ ATPase) family [27], as well as the *Kv* depolarisation-activated outward-rectifying potassium channels, paralogous to those involved in potassium release during an animal AP (although non-inactivating and displaying three orders of magnitude slower kinetics), are good candidates for the repolarisation of the cell [5,7,28]. Any identification of the molecular players involved in plant electrical signal propagation or decoding would therefore represent an advance, both for our understanding of electrical signalling mechanisms and for the diverse roles of ion channels in both plants and animals.

Despite many similarities, plant and animal APs are different phenomena, and particularly the three orders of magnitude difference in both the duration and the propagation speed might raise the question as to whether both rely on similar processes and molecular mechanisms. Indeed, such a slow signal does not exclude a very different mechanism for plant AP generation and propagation compared to animals. Such a mechanism may not rely on voltage-gated channels, but rather involve for instance, molecular diffusion of a signal molecule such as nitric oxide, second messenger calcium etc. Here, we challenge the hypothesis that plant APs rely on a voltage-dependent mechanism like their animal counterparts, where triggering, propagation and shaping are governed by calcium and/or sodium (for depolarisation) and potassium (for repolarisation) voltage-gated channels. We used the model plant *Arabidopsis thaliana* and tested the involvement of potassium voltage-gated channels homologous to the *Kv* animal channels that are responsible for the repolarisation in neurons. We targeted two vascular tissue-localised voltage-sensitive channels: the weakly-rectifying AKT2, which allows both uptake and release of potassium [29,30,31], and GORK, a depolarisation-activated outward channel [32]. Both channels were found to affect excitability in Arabidopsis, albeit, by different means.

## 2. Results

### 2.1. Electric Stimulation Induces Action Potentials in Arabidopsis

**Figure 1:**
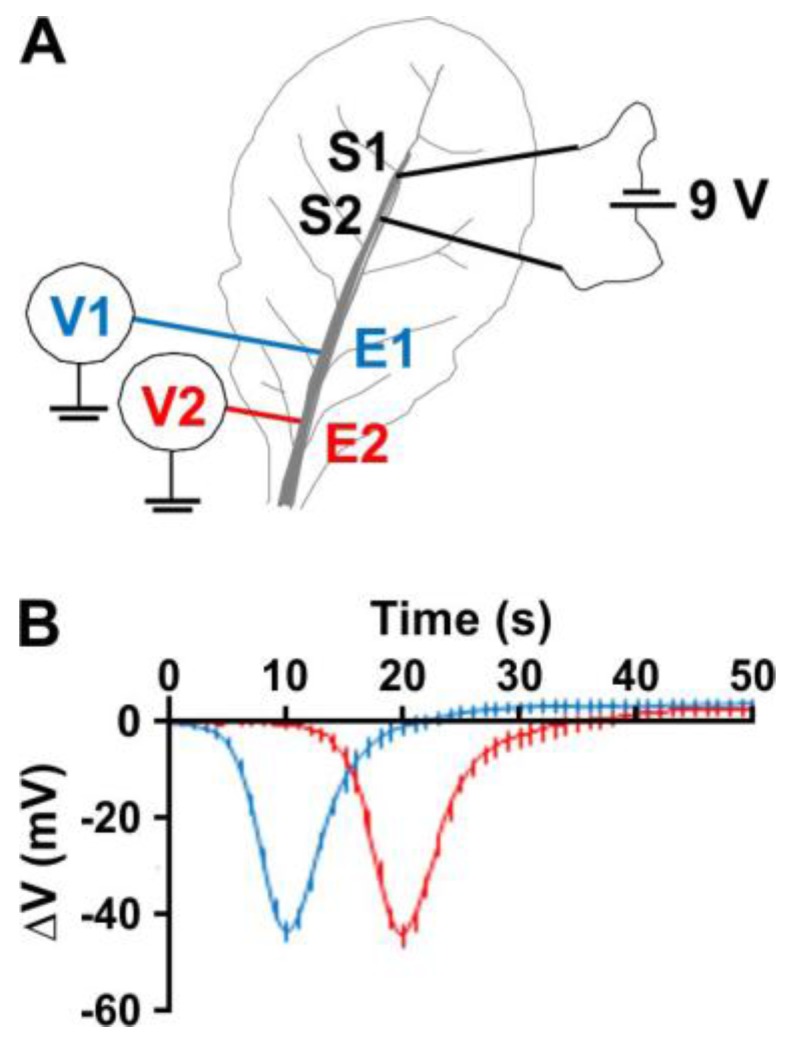
Action potentials directly recorded in Arabidopsis leaves. (**A**) Scheme of the experimental setup. (**B**) Time-course of averaged APs (voltage as a function of time) at electrodes E1 (blue) and E2 (red) recorded in the electrically-stimulated WS ecotype (Means ± SE, *n* = 25). Zero time: start of the 5-s electrical stimulation.

An AP is an electric signal characterised as a transient depolarisation that propagates along the membrane of connected cells. The all-or-nothing character of an AP implies that its properties do not depend on the strength, intensity and duration of the triggering stimulus. Along the propagating path, APs are auto-generated, meaning that an AP is formed place to place, the approaching electric signal is re-amplified from one site to another, and the AP further spreads [33]. Other important characteristics are the duration, amplitude and speed of propagation of the elicited APs, and the refractory period—the time required for the membrane to recover before a subsequent AP can be triggered. Among the different possibilities for eliciting an AP in plants, electric stimulation is often the method of choice [33,34,35,36,37]), primarily due to its high reproducibility. Favre and Degli Agosti have analysed the different parameters of APs in the model plant *Arabidopsis thaliana* and have determined stable conditions that allow the reproducible electric stimulation of APs [33]. Based on these findings, we applied for 5 s, an external electric field of 0.6 mV/μm through fine wire electrodes inserted into the vascular tissue of a leaf to elicit an AP in Arabidopsis (Figure 1). The response to the applied stimulus was recorded in WS and Col-0 wildtypes and showed the characteristic shape of a plant AP, a ~15-s-long bell shape pulse travelling at approximately 1 mm/s (Figure 1B). It did self-propagate through the leaf vascular tissue, as indicated by its stable amplitude (Figure 1). At no time did we record an electrical signal with the typical shape of a variation potential (i.e., a sharp depolarisation followed by a very slow repolarisation), a signal that does not self-propagate and diminishes away from the stimulus [17].

**Figure 2:**
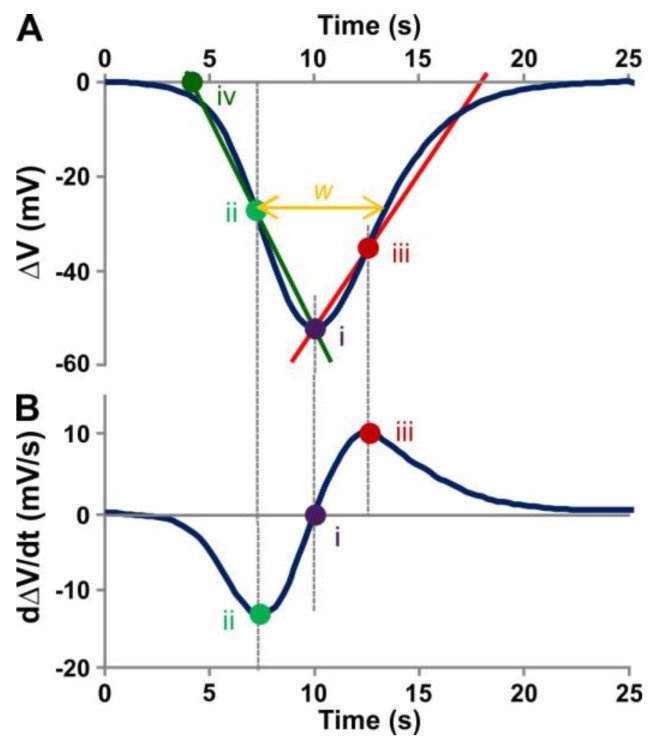
Analysis of action potentials. APs were recorded relative to the baseline, converted to a Microsoft excel file and imported into SigmaPlot (Version 11; Systat Software, San Jose, CA, USA). (**A**) For analyses, data were smoothed by calculating the running average over four neighbouring points using the “2 D smoothing function”. (**B**) dΔ*V*/d*t* values were then obtained using the “Compute 1st Derivative” function. To elaborate standardised characteristic parameters, an AP was approximated by a triangle shape (green and red lines). For this, the peak of the AP (Point i) as well as the reversal Points ii and iii (dΔ*V*/d*t* = 0), were determined. The intersection of the linear regression between Points i and ii with the time axis determined the start point of the AP (Point iv). The AP signal width (w) was measured as the change in time (Δ*t*) at the half-maximum AP amplitude. The propagation speed of the signal was taken from the measured distance between the two recording electrodes at E1 and E2, divided by the time in seconds between the two start points (Point iv) at E1 and E2.

To quantify the characteristic parameters of the measured APs, we determined their amplitude, velocity and width in a standardised procedure (Figure 2). The APs measured in both ecotypes did not differ significantly (*p* >0.05, *t*-test) in these parameters (Table 1). The signal amplitude was 47.2 ± 11.2 mV in WS and 52.0 ± 12.3 mV in Col-0, the propagation velocity was 1.3 ± 0.3 mm s^−1^ (WS) and 1.0 ± 0.2 mm s^−1^ (Col-0), and the signal width was 10.9 ± 4.2 s (WS) and 7.8 ± 2.5 s (Col-0). The two ecotypes differed, however, in their responsiveness. While in 88% of all tested Col-0 plants an AP could be elicited by the electric stimulation, WS plants were less excitable with a response rate of 51%. These results, including the ecotype-specificity, mirror those reported earlier by Favre and Degli Agosti [33] and further support the conclusion that the electric phenomena investigated here are true APs, not other electric responses.

### 2.2. The Outward-Rectifying Channel GORK Limits Membrane Depolarisation and Accelerates Its Repolarisation

**Figure 3:**
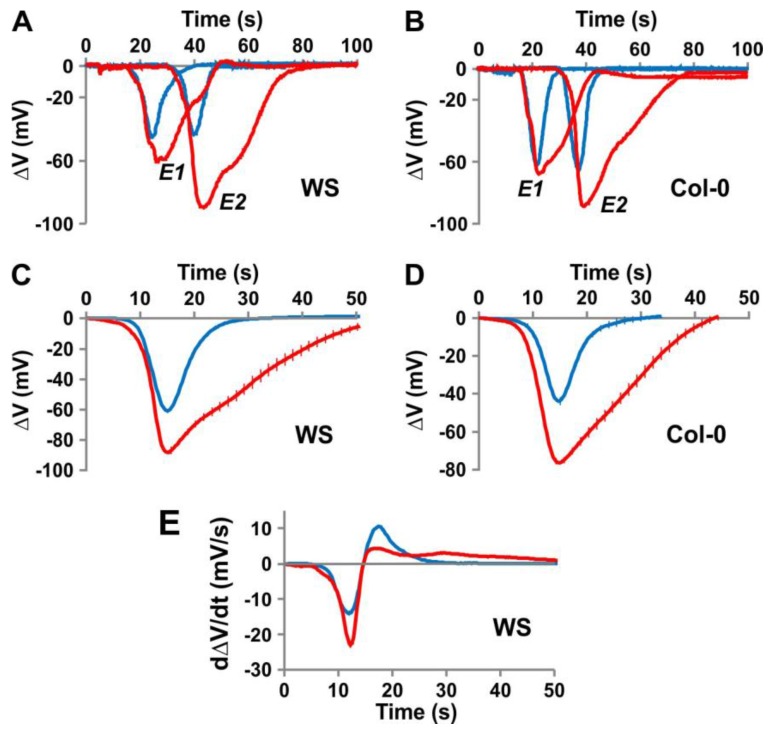
Dynamic characteristics of action potentials in wildtype compared to *gork* knockout mutants. (**A**,**B**). Time-course of typical APs (voltage as a function of time) recorded at electrodes E1 and E2 in WS wildtype ((**A**), blue) compared to that of the WS *gork* knockout ((**A**), red) and the Col-0 wildtype ((**B**), blue) compared to that of the *gork* knockout in the Col-0 background ((**B**), red). Zero time: start of the 5-s electrical stimulation (Figure 1A). (**C**) Time-course of averaged APs from the E2 recording electrode for the *gork* knockout mutant in the WS background (red) and the WS wildtype (blue) (*n* = 25). (**D**) Time-course of averaged APs from the E2 recording electrode for the *gork* knockout mutant in the Col-0 background (red) and the wildtype Col-0 (blue) (*n* = 25). (**E**) Time-course of averaged AP derivatives (dΔ*V*/d*t* as a function of time) from the E2 recording electrode for the *gork* knockout mutant in the WS background (red) and the wildtype WS (blue) (*n* = 25).

Plant APs are thought to be transmitted by electrical propagation through the phloem tissue [5,28]. In animal cells, voltage-gated potassium channels play a key role in APs, opening in response to membrane depolarisation so driving the voltage back to its resting potential [38]. Accordingly, we tested in Arabidopsis whether a depolarisation-activated channel, the potassium Shaker-like channel GORK has a similar role in repolarisation. We monitored APs in response to electrical stimuli in *gork* knockout plants from two ecotypes (Col-0 and WS) and observed a strong electrical phenotype in both WS and Col-0 *gork* plants compared to their respective wildtype (Figure 3). The *gork* mutation affects the whole shape of the signal, both the depolarisation and repolarisation phases (Figure 3A), and influences the ability of the plant to generate an AP (Table 1). In addition, we noted that knocking out the gene results in an augmentation of the signal amplitude as it travels through the leaf (Figure 3). This amplification is observed in the *gork* plants in both the Col-0 and WS backgrounds (Figure 3A,B, Table 1) and could suggest a difference between the leaf blade and the blade to petiole transition zone, which we have so far been unable to identify.

Both the speed and degree of the depolarisation were greater in *gork* knockout lines than in wildtype, whilst the speed of the repolarisation was much slower. Closer analysis of the repolarisation phase of the derivative curve is particularly informative. It is much faster in wildtype (up to 10 mV/s) than in *gork* (<5 mV/s), suggesting that GORK, a strongly voltage-gated channel, is the main repolarising actor in the wildtype. In the *gork* mutant, repolarisation has to be achieved by a weaker voltage-sensitive transporter such as the proton pump.

### 2.3. The Weakly-Rectifying Shaker-Like Channel AKT2 Affects Plant Excitability by Virtue of Its Rectification Mode

In animal cells, background non-rectifying potassium channels regulate membrane excitability by clamping the membrane voltage to the potassium equilibrium potential and decreasing cell excitability [38]. The Arabidopsis K_weak_ channel AKT2 is an inward-rectifying potassium channel, which can be converted into a non-rectifying channel that is open over the entire physiological voltage-range by a cascade of post-translational modification steps [39,40]. We examined any involvement of AKT2 in APs in transgenic Arabidopsis plants expressing modified AKT2 channels: (i) The WS *akt2-1* knockout mutant (*akt2-1*) and the Col-0 *akt2-2* knockout mutant; (ii) AKT1-NN, expressing the mutated channel AKT2-S210N-S329N in the *akt2-1* background. AKT2-S210N-S329N is more easily converted into the non-rectifying mode than the wild type; (iii) AKT2-AA, expressing the mutated channel AKT2-S210A-S329A in the *akt2-1* background. This mutant cannot be converted into the non-rectifying mode—it is only inward-rectifying; (iv) AKT2-KS, expressing the mutant channel AKT2-K197S, which also functions solely as an inward-rectifying channel [39,41,42,43]. No differences in either the speed or the width of the generated signal were observed between any of the mutant plants or between mutants and the respective wildtypes (Table 2). However, plant excitability, defined as the fraction of plants displaying an AP after electrical stimulation, is affected in the different genotypes (Table 2), with AKT2-KS = AKT2-AA < *akt2-1* < wildtype < AKT2-NN for the WS ecotype and *akt2-2* < wildtype for the Col-0 ecotype (significant at the 95% confidence limit from Bernoulli trials). Thus, the loss of the AKT2 channel in the open configuration, as in the *akt2-1* and *akt2-2* knockout plant and in the AKT2-KS, AKT2-AA mutant plants, reduced plant excitability. It might be speculated that the different rectification properties of the AKT2 channel in the three genetic backgrounds are likely to affect extracellular potassium levels (apoplastic [K^+^]). An open channel (AKT2-NN) could result in higher apoplastic [K^+^], and an inward rectifier (AKT2-KS), in lower apoplastic [K^+^]. AKT2 channels could therefore have an effect on membrane excitability by controlling both the electrical membrane properties and the apoplastic potassium concentration.

## 3. Discussion

In this study we used an established, albeit coarse technique to induce APs in Arabidopsis. We required a reliable, AP-inducing stimulus that would allow consistent and repeatable measurements, necessary for screening for excitability in different mutant lines. Therefore, we followed a procedure that has been shown in a previous publication [33] to provoke a response in 60 to 80% of Arabidopsis Col-0 plants. It should be noted that the applied stimulus of 0.6 mV/μm for about 5 s was much smaller than that applied in other studies. Tomato leaves, for instance, were challenged with stimuli as high as 30 V and no any damage in the leaf around the simulated zone was observed [35]. In line with this finding of robustness, the plants in our study did not show any sign of damage. Plants were able to elicit a further signal in the same leaf with the electrodes unmoved from the previous stimulus 6 h after the initial stimulation.

### 3.1. Plant APs Could Be Carried by Anions and K^+^

Early studies on APs in sensitive plants such as the Venus flytrap [44] or in Characean algae suggested that the associated ion fluxes differ from those of animal electrical signals. Animal APs are generally induced by sodium influx; only in certain cases are they initiated by calcium, for example myocytes [38]. Repolarisation of animal APs results from a potassium efflux through *Kv* channels [38]. In plants, the depolarisation is proposed to be induced by anion fluxes, and the repolarisation, as in animals, by potassium [3,9,11,12,13,14,16]. The results obtained with the *gork* knockout plants (Figure 3) strongly support the hypothesis of depolarisation-activated K^+^ channels being involved in plant AP propagation and modulation. A physiological role for the GORK potassium channel was first proposed in guard cells [45]. There, GORK was described as a modulator of intracellular potassium homeostasis, mediating potassium depletion of guard cells during stomatal closure. The results presented here indicate that GORK could have a broader physiological role through the control of the membrane potential. To support this conclusion, we modelled plant APs in a computational cell biology approach.

### 3.2. Computational Simulation of Plant AP Generation and Propagation Allows Mechanistic Insight into the Contribution of GORK

**Figure 4:**
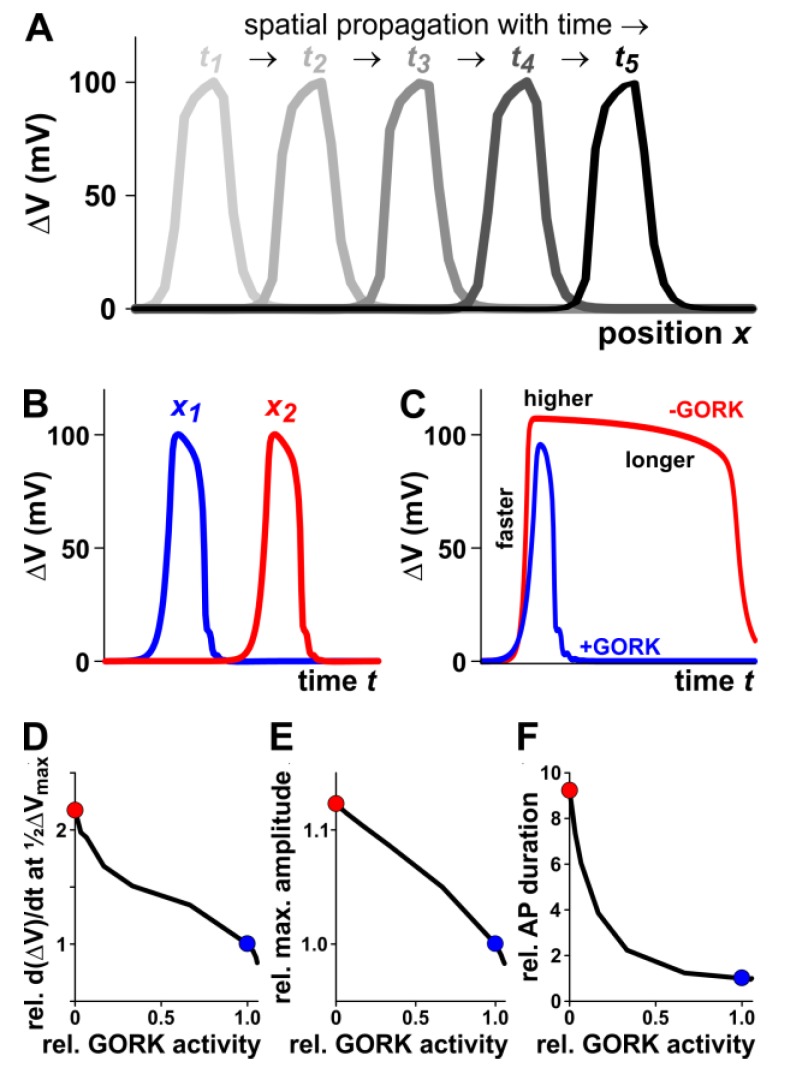
Computational simulation of plant action potentials displayed in a qualitative manner. (**A**) An action potential induced by a short electrical stimulus then propagates in a self-amplifying manner along the ‘green cable’. Displayed is the calculated membrane voltage for a segment of the cable for five different time points (different grey shades). (**B**) At two different positions *x*_1_ (blue curve) and *x*_2_ (red curve) the APs pass at different times. (**C**) Effect of the presence (blue curve) or absence (red curve) of the K^+^ conductance of the GORK channel. A ‘green cable’ without GORK channels has APs that rise faster, reach a higher maximal amplitude and last longer. (**D**) Dependency of the steepness of the initial phase of an AP on the activity of the repolarising K^+^ conductance. (**E**) Dependency of the maximal amplitude of an AP on the activity of the repolarising K^+^ conductance. (**F**) Dependency of the duration of an AP on the activity of the repolarising K^+^ conductance. The red and blue dots in (**D**–**F**) represent the two situations displayed in (**C**).

An AP propagating along the ‘green cable’ can be simulated in a simplified manner with (i) an H^+^-ATPase-driven background conductance, (ii) an anion channel, which rapidly activates at voltages more positive of −60 mV and then slowly inactivates and (iii) a K^+^ channel, which activates less rapidly than the anion channel at voltages positive of E_K_ (Figure 4A and Appendix A). It should be noted that the sign of the amplitude and the detailed shape of the simulated APs in the repolarisation phase in particular, differ from the APs measured in our study. However, this is not surprising because in the simulation, we calculate the membrane voltage propagating along the plasma membrane, while in our experimental approach we measured extracellular potentials (as previously done in animal cells [46,47]). Important at this stage is the self-propagating feature of the signals in both cases. And indeed, a stimulus applied for a short time at one edge induces an AP that moves along the ‘green cable’ (Figure 4A). At two different points along the cable, the AP is registered at different times (Figure 4B, compare with Figure 1).

When we repeated the simulations without GORK channels, we realised three essential qualitative changes of the AP: (i) the electrical signal reaches its maximal value faster, (ii) the maximal amplitude is higher, and (iii) the depolarisation phase is longer (Figure 4C). This was also observed experimentally when comparing APs from wildtype and *gork* knockout plants (Figure 3). The contribution of the GORK channel to the shape of the plant AP was further substantiated by simulating the APs not only in the presence and absence of GORK, but also with different GORK activity levels. The reduction of the GORK activity speeds up the initial depolarisation (Figure 4D; slope of the ΔV(*t*) curve at half-maximal depolarisation), it increases the maximal amplitude of the AP (Figure 4E), and it prolongs the depolarisation (Figure 4F). Interestingly, if we increased the GORK activity, we reached a threshold above which we lost excitability in our modelled system.

All the modelled data are qualitatively fully in line with the experimental observations and strongly support the hypothesis that GORK dominates the repolarisation of the membrane during a plant AP. We suggest that GORK repolarises the membrane using the potassium battery energy source [48]. In the *gork* knockout, repolarisation is likely driven by energy expensive H^+^-ATPases, which have to overcome the anion (and/or Ca^2+^) and other unknown background conductances. These determine the fine-tuned shape of the repolarisation phase in the absence of GORK. For our model, we unfortunately had insufficient information about the conductances that could provide a further explanation of the differences in the exact shape of the repolarisation phase between the model and experiment. Nevertheless, the qualitative changes allow fundamental mechanistic insights into different roles of GORK in plant APs: GORK not only contributes towards the end of an AP by repolarising the membrane (Figure 4F) but also influences the beginning. At the onset of an AP, anion (or Ca^2+^) channels are activated, but a tiny although significant amount of GORK channels is also stimulated in this early phase. This initial GORK activity dampens the rapid depolarisation (smaller slope; Figure 4D) and caps the maximum amplitude reached (Figure 4E).

### 3.3. Mechanism of Plant AP Propagation—Likely Not a Pure Electric Phenomenon

The very basic model presented in the last paragraph explains in a straightforward manner, the principle role of depolarisation-activated K^+^ channels in plant APs. This model, however, has not yet taken into account two additional essential features: (1) an AP is accompanied by a significant efflux of K^+^, which likely alters the apoplastic K^+^ concentration when an AP is passing by, and (2) channels of the GORK-type sense the external K^+^ concentration [49]; thus, they react on the presumed K^+^-wave. The mathematical handling of these effects would go far beyond the scope of this article and will be tackled in future work. However, the consequences of the feedback processes can already be understood on the basis of the presented rudimentary model.

An interesting finding from our modelling approach is that there is a threshold of the GORK activity, above which we lost excitability in our model system. The investigation as to why such a threshold exists, revealed that in this case, the outward K^+^ currents are already too large to be overrun by the electric signal when the stimulus arrives. In fact, under these conditions, there is a stable equilibrium at moderate negative voltages at which the steady state K^+^ efflux through residual active GORK channels is electrically compensated by an anion efflux. An incoming electric stimulus can only briefly disturb this equilibrium, but not trigger the firing of an AP because of the stabilising effect of the GORK channel. However, if the electric pulse is accompanied by an apoplastic K^+^-wave caused by the GORK channels opened during an AP, the increasing external potassium concentration in the closer environment (i) increases E_K_ and (ii) shifts the activation threshold of GORK to less negative voltages, thus reducing its open probability [49]. Both effects are equivalent to a reduction of the GORK activity, which in turn, increases in not yet stimulated regions, the excitability of the system. Thus, the presumed potassium wave would prime the ‘green cable’ from a stable, non-excitable state into a bi-stable, excitable state. 

It is thus tempting to speculate that plant APs are not of pure electric nature but instead are a combination of an electrical component and one or more chemical components, with potassium being critical. This concept would explain the slow speed of APs in plants, which then would be limited by the diffusion of the chemical component(s). Interestingly, this concept would also explain the observations in the experiments with AKT2 (Table 2). As speculated above, AKT2 in the open channel conformation could be responsible for a higher apoplastic [K^+^], which would down-regulate GORK activity and increase excitability. The elimination of AKT2 would eliminate this effect and would, as observed, reduce excitability. The conversion of AKT2 into a pure K^+^-uptake channel (AKT2-KS, and AKT2-AA) would favour the sequestration of K^+^ by the cells and thus reduce apoplastic [K^+^]. This would, again as observed, further reduce excitability.

### 3.4. Integration into Plant Physiology

Plant and animal APs may have different natures, but they have a similar role: long-distance signalling. Interestingly, the same molecules (Shaker channels as well as glutamate receptors) generate and propagate APs in both systems. Electrical signals (and/or their underlying molecular effectors) are proposed as integral components of the jasmonate (JA)-signalling pathway in plants [19]. Interestingly, transcriptional and physiological links have been shown between the JA-pathway (involved in biotic stress responses), and the abscisic acid (ABA)-pathway (a primary abiotic stress-activated hormone) [50,51]. Animal APs are frequently regulated by hormones and neurotransmitters, which modulate potassium channel activity. It is tempting to speculate that in plants, hormones such as ABA play a similar role. This would allow the activation of the JA-pathway in response to abiotic stress. Indeed, both AKT2 and GORK channels play important roles in plant stress responses [41,45], in particular, in the ABA-dependent signalling cascade involved in the response to abiotic stress. ABA regulates both *AKT2* [52] and *GORK* [32] activities, and in tomato, the ability of a plant to trigger an AP is dependent on the ABA concentration [53]. Therefore, through ABA-dependent regulation, AKT2 and GORK, along with the already identified GLRs [19], could modulate AP signals and the downstream JA pathway. More generally, APs could be integrated with several different signalling pathways. Indeed, waves of both reactive oxygen species [54,55] and calcium [55,56] move through an Arabidopsis plant at similar speeds to those we report for APs. Understanding such signalling networks is vital to our attempts to comprehend how plants can adapt to and tolerate adverse environmental conditions.

## 4. Materials and Methods

### 4.1. Plant Material

*Arabidopsis thaliana* (L.) ascensions Columbia (Col-0) and Wassilewskija (WS) wildtypes, *akt2-2* [57] and *akt2-1* [58] knockout lines in the Col-0 and WS backgrounds, respectively, insertion lines of a modified AKT2 channel in the WS *akt2-1* background [39,41], and *gork* knockout lines in the Col-0 and WS backgrounds [45,59] were grown in soil under 8 h/16 h day/night conditions. 38 to 42 day old plants were used for recording electrical signals.

### 4.2. Arabidopsis Leaf with Stimulation and Recording Electrodes

All measurements were undertaken in a Faraday cage. Two stimulating Ag/AgCl electrodes (S1, S2) (Figure 1), connected via thin coated copper wires to a 9 Volt battery, were inserted into the mid-vein of a leaf, towards the leaf apex, 15 mm apart. The two recording electrodes (E1, E2) were inserted into the mid-vein of the same leaf, the distal one, E2, at the beginning of the petiole and the proximal, E1, halfway between S2 and E2. E1 and E2 were connected with thin-coated copper wires to an Analogue/Digital converter (National Instruments, USB-6225, Austin, TX, USA), which delivered, at a sampling rate of 5 Hz, numerical values of E1 and E2 voltages in differential mode with respect to the voltage of an Ag/AgCl reference electrode inserted into the soil. Six hours after installation, an electrical stimulation was applied by closing the connection between S2 and the negative pole of the battery for 5 s. The outputs of the A/D interface were displayed on a computer screen and stored on a hard disk for subsequent analyses. Characteristic parameters of the APs were analysed as described in Figure 2.

### 4.3. Modelling Approach

To qualitatively illustrate the contribution of outward-rectifying K^+^ channels to plant APs, we computationally modelled APs in the ‘green cable’ ([28]; for technical details see Appendix A) by the interplay of three basic electrogenic transporters. (i) the H-ATPase-driven background conductance was mathematically described by I_background_ = i_BGmax_ × (1 − exp[−0.1 × (V + 70 mV)/25 mV])/(1 + exp[−0.1 × (V + 70 mV)/25 mV]) ≈ g_BG_ × (V + 70 mV) [60], with the parameter φ_BG_ = g_BG_/C_M_ = 1 × s^−1^. (ii) Because plant outward-rectifying K^+^ channels do not show any sign of C- or N-type inactivation even under prolonged stimulation, they can be described in a first approximation by a two-state model (open and closed [49]), with activation and deactivation rate constants of a_K_ = exp(V/25 mV – 3.0) × s^−1^ and d_K_ = exp(−0.6 × V/25 mV – 2.0) × s^−1^, respectively. The Nernst equilibrium for K^+^ was E_K_ = −70 mV and the K^+^-conductance-dependent parameter was φ_K_ = g_K_/C_M_ varied between 31.7 × s^−1^ (limit value for which still propagating APs can be induced) and 0 × s^−1^ (no K^+^ channels). (iii) A depolarisation-activated, slowly inactivating anion (or Ca^2+^) channel was described by a four-state model (open, open-inactivated, closed, closed-inactivated), with activation and deactivation rate constants of a_A_ = exp(V/25 mV – 1.0) × s^−1^ and d_A_ = exp(−1.4 × V/25 mV + 1.4) × s^−1^, and inactivation and recovery rate constants of in_A_ = exp(0.2 × V/25 mV – 3.0) × s^−1^ and re_A_ = exp(−3.0 × V/25 mV – 6.0) × s^−1^, respectively. The Nernst equilibrium for the depolarising ion was E_A_ = +40 mV and the conductance-dependent parameter was φ_A_ = g_A_/C_M_ = 120 × s^−1^. Simulations were carried out numerically as described in the Appendix for 200 points along the *x*-axis (matrix index *n*) and 5001 time-points (matrix index *m*) with time interval of Δ*t* = 10 ms and α = g_x_/C_M_ × Δ*t*/(Δ*x*)^2^ = 0.02. Action potentials were induced by a stimulus $\epsilon_{n}^{m}=10\;\mathrm{mV}$ at position *n* = 1 for 499 time-intervals Δ*t* (*m* = 1, …, 500).

## Figures and Tables

**Table 1:** Numerical data for action potentials (APs) recorded in electrically-stimulated wildtype and *gork* knockout mutants of WS and Col-0 ecotypes. *gork* knockout mutants were compared to their respective wildtype (WT). Excitability was taken as the number of plants (of the 100 tested) displaying an AP after electrical stimulation. For other data, 25 plants displaying typical APs were used (Means ± SD). Because APs dramatically increased upon propagation in *gork* knockout mutants, signals recorded by both E1 and E2 were considered separately for amplitude and width statistics. Speed of propagation was the distance between E1 and E2 divided by the time-shift between APs recorded at these electrodes.

	WS Ecotype		Col-0 Ecotype	
	WT	*gork*	WT	*gork*
Excitability (%)	51%	59%	88%	73%
Amplitude, E1 (mV)	44.6 ± 10.4	50.4 ± 13.6	58.3 ± 5.9	63.8 ± 21.8
Amplitude, E2 (mV)	47.2 ± 11.2	81.8 ± 8.9	62.2 ± 5.5	93.6 ± 13.3
Width, E1 (s)	6.7 ± 1.7	13.6 ± 3.4	6.6 ± 2.4	13.6 ± 6.4
Width, E2 (s)	6.7 ± 1.6	18.1 ± 5.0	7.6 ± 1.5	19.7 ± 6.9
Speed (mm s^−1^)	1.3 ± 0.3	1.1 ± 0.6	1.2 ± 0.4	1.2 ± 0.3

**Table 2:** Numerical data for action potentials (APs) recorded in electrically-stimulated wildtype and *AKT2* mutants of WS and Col-0 ecotypes. Knockout mutants were compared to their respective wildtype (WT), *akt2-1* for WS and *akt2-2* for Col-0. The other studied AKT2 mutant lines were all of the WS ecotype, expressing, in the WS *akt2-1* knockout background, modified AKT2 channels carrying either a single K198S substitution (WS AKT2-KS), double substitutions S210A-S329A (WS AKT2-AA) or S210N-S329N (WS AKT2-NN). Excitability was taken as the number of plants (of the 100 tested) displaying an AP after electrical stimulation, as recorded at electrodes 1 and 2 (E1 and E2, see Figure 1A). From each genotype, 25 plants that displayed typical APs were used for amplitude and width statistics (see Figure 2 for method), using only APs recorded by E2. The speed of propagation is the distance between E1 and E2, divided by the time-shift between APs recorded at these electrodes. (Means ± SD, *n* = 25).

Line	WS Ecotype					Col-0 Ecotype	
	AKT2-KS	AKT2-AA	*akt2-1*	WT	AKT2-NN	*akt2-2*	WT
Excitability (%)	26%	31%	41%	51%	59%	63%	88%
Amplitude (mV)	43.2 ± 14.5	41.0 ± 7.3	38.1 ± 10.6	47.2 ± 11.2	53.0 ± 9.2	52.0 ± 12.3	62.2 ± 5.5
Width (s)	7.8 ± 2.8	7.7 ± 1.9	6.6 ± 2.1	10.9 ± 4.2	6.7 ± 1.6	7.8 ± 2.5	7.8 ± 1.5
Speed (mm/s)	0.9 ± 0.3	1.3 ± 0.4	1.2 ± 0.4	1.3 ± 0.3	1.1 ± 0.4	1.0 ± 0.2	1.2 ± 0.4

## Appendix A. Computational Simulation of Action Potentials Using the Cable Equation

The basis of a propagating electric signal in a biological cable is the cable equation:

(A1) $$\frac{\partial }{\partial t}V\left(t,x\right)=\frac{g_{x}}{C_{M}}\cdot \frac{\partial^{2}}{\partial x^{2}}V\left(t,x\right)+\frac{1}{C_{M}}\left[J_{Stim}\left(t,x\right)-\underset{i}{\sum }g_{i}\cdot p_{i}\left(t,x,V\left(t,x\right)\right)\cdot \left[V\left(t,x\right)-E_{i}\right]\right]$$

Here, the different parameters mean: (1) *V*(*t*,*x*): Voltage at position *x* at time *t*; unit: mV; (2) *i*: ion species to be considered; in Neurons: K^+^ and Na^+^; in plants, H^+^, K^+^ and Cl^−^. (3) $E_{i}$: equilibrium voltage for ion species *i*; unit: mV; (4) $g_{i}$: maximum conductance for ion species *i*; unit: pS·µm^−2^; (5) $p_{i}\left(t,x,V\left(t,x\right)\right)$: “channel open probability” = activity of the conductance; $0\leq p_{i}\leq 1$; (6) $J_{stim}$: external stimulus to excite the system; unit: fA·µm^−2^; (7) $C_{M}$: specific membrane capacity; unit: pF·µm^−2^; (8) $g_{x}$: conductance along the *x*-axis; unit: pS.

By the following re-definitions, the redundancy in system-specific parameters can be removed: (1) $\varphi_{x}=\frac{g_{x}}{C_{M}}$ (unit: µm^2^·s^−1^) is a system specific constant. A large $\varphi_{x}$ means a large $g_{x}$ or a small $C_{M}$, which is equivalent to a faster propagation, while a small $\varphi_{x}$ means a small $g_{x}$ or a large $C_{M}$ and a slower propagation. (2) $\epsilon_{Stim}\left(t,x\right)=\frac{J_{Stim}\left(t,x\right)}{C_{M}}$ (unit: mV·s^−1^) describes the applied external stimulus. (3) $\varphi_{i}=\frac{g_{i}}{C_{M}}$ (unit: s^−1^) are channel-specific constants, which depend on the number of channels and the single channel conductance. With these definitions, the cable equation is:

(A2) $$\frac{\partial }{\partial t}V\left(t,x\right)=\varphi_{x}\cdot \frac{\partial^{2}}{\partial x^{2}}V\left(t,x\right)+\epsilon_{Stim}\left(t,x\right)-\underset{i}{\sum }\varphi_{i}\cdot p_{i}\left(t,x,V\left(t,x\right)\right)\cdot \left[V\left(t,x\right)-E_{i}\right]$$

A practical approach to solve the cable equation numerically is the Crank-Nicolson method. Here, the differentials are approximated by differences:

(A3) $$\frac{\partial }{\partial t}V\left(t,x\right)\to \frac{V\left(t+\Delta t,x\right)-V\left(t,x\right)}{\Delta t}$$

(A4) $$\frac{\partial^{2}}{\partial x^{2}}V\left(t,x\right)\to \frac{1}{2{\left(\Delta x\right)}^{2}}\left[\begin{array}{c} V\left(t+\Delta t,x+\Delta x\right)-2\cdot V\left(t+\Delta t,x\right)+V\left(t+\Delta t,x-\Delta x\right)\\ +V\left(t,x+\Delta x\right)-2\cdot V\left(t,x\right)+V\left(t,x-\Delta x\right) \end{array}\right]$$

(A5) $$V\left(t,x\right)\to \frac{1}{2}\cdot \left[V\left(t+\Delta t,x\right)+V\left(t,x\right)\right]$$

Additionally, the *x*-dimension is discretized in *N* points (index *n* = 0, …, *N*) with the distance of Δx between two neighbouring points; and the *t*-Dimension is discretized in *M* points (index *m* = 0, …, *M*) with the interval of Δt between two neighbouring points: $V_{n}^{m}=V\left(t,x\right)$; $V_{n+1}^{m}=V\left(t,x+\Delta x\right)$; $V_{n-1}^{m}=V\left(t,x-\Delta x\right)$; $V_{n}^{m+1}=V\left(t+\Delta t,x\right)$; $V_{n+1}^{m+1}=V\left(t+\Delta t,x+\Delta x\right)$; $V_{n-1}^{m+1}=V\left(t+\Delta t,x-\Delta x\right)$; $pi_{n}^{m}=p_{i}\left(t,x,V\left(t,x\right)\right)$.

With

(A6) $$\alpha =\frac{\varphi_{x}\cdot \Delta t}{{\left(\Delta x\right)}^{2}},$$

(A7) $$a_{n}^{m}=\Delta t\cdot \sum_{i}\varphi_{i}\cdot pi_{n}^{m},$$

(A8) $$b_{n}^{m}=2\Delta t\cdot \sum_{i}\varphi_{i}\cdot pi_{n}^{m}\cdot E_{i},$$

(A9) $$\epsilon_{n}^{m}=2\Delta t\cdot \epsilon_{Stim}\left(t,x\right)$$

the differential equation converts into a linear equation system:

(A10) $$\left[A^{m}\right]\cdot {\vec{V}}^{m+1}=\left[B^{m}\right]\cdot {\vec{V}}^{m}+{\vec{\epsilon }}^{m}+{\vec{b}}^{m}$$

with the matrices

(A11) $$\mathrm{diag}\left[{\vec{a}}^{m}\right]=\left(\begin{array}{cccc} a_{1}^{m} & 0 & \ldots & 0\\ 0 & a_{2}^{m} & \ldots & 0\\ \vdots & \vdots & \ddots & \vdots \\ 0 & 0 & \ldots & a_{N}^{m} \end{array}\right),$$

(A12) $$\left[A^{m}\right]=\left[A\right]+\mathrm{diag}\left[{\vec{a}}^{m}\right],$$

(A13) $$\left[B^{m}\right]=\left[B\right]-\mathrm{diag}\left[{\vec{a}}^{m}\right]$$

(A14) $$\left[A\right]=\left[\begin{array}{ccccccc} 2+2\alpha & -\alpha & 0 & & & & \\ -\alpha & 2+2\alpha & -\alpha & \ldots & & 0 & \\ 0 & -\alpha & 2+2\alpha & & & & \\ & \vdots & & \ddots & & \vdots & \\ & 0 & & \ldots & -\alpha & 2+2\alpha & -\alpha \\ & & & & 0 & -\alpha & 2+2\alpha \end{array}\right]$$

(A15) $$\left[B\right]=\left[\begin{array}{ccccccc} 2-2\alpha & \alpha & 0 & & & & \\ \alpha & 2-2\alpha & \alpha & \ldots & & 0 & \\ 0 & \alpha & 2-2\alpha & & & & \\ & \vdots & & \ddots & & \vdots & \\ & 0 & & \ldots & \alpha & 2-2\alpha & \alpha \\ & & & & 0 & \alpha & 2-2\alpha \end{array}\right]$$

and the vectors

(A16) $${\vec{V}}^{m+1}=\left(\begin{array}{c} V_{1}^{m+1}\\ \vdots \\ V_{n-1}^{m+1}\\ V_{n}^{m+1}\\ V_{n+1}^{m+1}\\ \vdots \\ V_{N}^{m+1} \end{array}\right),$$

(A17) $${\vec{V}}^{m}=\left(\begin{array}{c} V_{1}^{m}\\ \vdots \\ V_{n-1}^{m}\\ V_{n}^{m}\\ V_{n+1}^{m}\\ \vdots \\ V_{N}^{m} \end{array}\right),$$

(A18) $${\vec{\epsilon }}^{m}=\left(\begin{array}{c} \epsilon_{1}^{m}\\ \vdots \\ \epsilon_{n-1}^{m}\\ \epsilon_{n}^{m}\\ \epsilon_{n+1}^{m}\\ \vdots \\ \epsilon_{N}^{m} \end{array}\right)$$

(A19) $${\vec{a}}^{m}=\left(\begin{array}{c} \Delta t\cdot \sum_{i}\varphi_{i}\cdot pi_{1}^{m}\\ \vdots \\ \Delta t\cdot \sum_{i}\varphi_{i}\cdot pi_{n-1}^{m}\\ \Delta t\cdot \sum_{i}\varphi_{i}\cdot pi_{n}^{m}\\ \Delta t\cdot \sum_{i}\varphi_{i}\cdot pi_{n+1}^{m}\\ \vdots \\ \Delta t\cdot \sum_{i}\varphi_{i}\cdot pi_{N}^{m} \end{array}\right),$$

(A20) $${\vec{b}}^{m}=\left(\begin{array}{c} 2\Delta t\cdot \underset{i}{\sum }\varphi_{i}\cdot pi_{1}^{m}\cdot E_{i}\\ \vdots \\ 2\Delta t\cdot \underset{i}{\sum }\varphi_{i}\cdot pi_{n-1}^{m}\cdot E_{i}\\ 2\Delta t\cdot \underset{i}{\sum }\varphi_{i}\cdot pi_{n}^{m}\cdot E_{i}\\ 2\Delta t\cdot \underset{i}{\sum }\varphi_{i}\cdot pi_{n+1}^{m}\cdot E_{i}\\ \vdots \\ 2\Delta t\cdot \underset{i}{\sum }\varphi_{i}\cdot pi_{N}^{m}\cdot E_{i} \end{array}\right)$$

After setting an appropriate value for α, the values of ${\vec{V}}^{m}$ can be obtained by iteratively, numerically solving the equation:

(A21) $${\vec{V}}^{m+1}={\left[A^{m}\right]}^{-1}\cdot \left\{\left[B^{m}\right]\cdot {\vec{V}}^{m}+{\vec{\epsilon }}^{m}+{\vec{b}}^{m}\right\}$$

**Algorithms**
- Define α, Δt, all $\varphi_{i}$, all *E_i_*, - ${\vec{\epsilon }}^{m}$ is pre-defined for all time points *m*; most entries are 0. - Iteration: ○ starting with ${\vec{V}}^{1}=V\left(0,x\right)$, ${\vec{pi}}^{1}=pi\left(0,x,V\left(0,x\right)\right)$ ○ *m* = 1, …, *M* − 1 ▪ calculate ${\vec{b}}^{m}$, ${\vec{a}}^{m}$, $\left[A^{m}\right]$, ${\left[A^{m}\right]}^{-1}$ and $\left[B^{m}\right]$ ▪ calculate ${\vec{V}}^{m+1}$ ▪ calculate ${\vec{pi}}^{m+1}$ via separate linear equation systems of the type: ▪ *n* = 1…*N* - *i* = 1, …, T (number of transporters involved) ○ $pi_{n}^{m+1}=pi_{n}^{m}+\Delta t\cdot \left[ai\left(V_{n}^{m}\right)\cdot \left(1-pi_{n}^{m}\right)-di\left(V_{n}^{m}\right)\cdot pi_{n}^{m}\right]$ with the transporter-specific coefficients $di\left(V_{n}^{m}\right)$ and $ai\left(V_{n}^{m}\right)$, which depend only on $V_{n}^{m}$ - End *i*-loop ▪ End *n*-loop ○ End *m*-loop - End Iteration
